# The potential of diagnostic point‐of‐care tests (POCTs) for infectious and zoonotic animal diseases in developing countries: Technical, regulatory and sociocultural considerations

**DOI:** 10.1111/tbed.13880

**Published:** 2020-10-30

**Authors:** Emma C. Hobbs, Axel Colling, Ratna B. Gurung, John Allen

**Affiliations:** ^1^ Australian Centre for Disease Preparedness (ACDP, formerly AAHL) Commonwealth Scientific and Industrial Research Organisation (CSIRO) East Geelong VIC Australia; ^2^ National Centre for Animal Health, Department of Livestock, Ministry of Agriculture and Forests Royal Government of Bhutan Thimphu Bhutan

**Keywords:** communicable disease control, developing countries, livestock, point‐of‐care testing, public health, validation and quality control, zoonoses

## Abstract

Remote and rural communities in low‐ and middle‐income countries (LMICs) are disproportionately affected by infectious animal diseases due to their close contact with livestock and limited access to animal health personnel). However, animal disease surveillance and diagnosis in LMICs is often challenging, and turnaround times between sample submission and diagnosis can take days to weeks. This diagnostic gap and subsequent disease under‐reporting can allow emerging and transboundary animal pathogens to spread, with potentially serious and far‐reaching consequences. Point‐of‐care tests (POCTs), which allow for rapid diagnosis of infectious diseases in non‐laboratory settings, have the potential to significantly disrupt traditional animal health surveillance paradigms in LMICs. This literature review sought to identify POCTs currently available for diagnosing infectious animal diseases and to determine facilitators and barriers to their use and uptake in LMICs. Results indicated that some veterinary POCTs have been used for field‐based animal disease diagnosis in LMICs with good results. However, many POCTs target a small number of key agricultural and zoonotic animal diseases, while few exist for other important animal diseases. POCT evaluation is rarely taken beyond the laboratory and into the field where they are predicted to have the greatest impact, and where conditions can greatly affect test performance. A lack of mandated test validation regulations for veterinary POCTs has allowed tests of varying quality to enter the market, presenting challenges for potential customers. The use of substandard, improperly validated or unsuitable POCTs in LMICs can greatly undermine their true potential and can have far‐reaching negative impacts on disease control. To successfully implement novel rapid diagnostic pathways for animal disease in LMICs, technical, regulatory, socio‐political and economic challenges must be overcome, and further research is urgently needed before the potential of animal disease POCTs can be fully realized.

## 1 INTRODUCTION

In many low‐ and middle‐income countries (LMICs), accurate and timely diagnosis of infectious diseases in human, let alone animal, populations remains challenging. Infectious agricultural and zoonotic diseases do not respect wealth or borders, and outbreaks of novel, re‐emerging and transboundary animal diseases can have devastating impacts on individual, community, regional and even global levels—as has become all too apparent with the ongoing African swine fever (ASF) epidemic, and the global COVID‐19 pandemic that is currently upending the lives and livelihoods of billions of people worldwide.

Livestock and poultry provide food, agricultural labour, clothing, fertilizers and social status and act as wealth assets to more than 600 million livestock‐dependent people around the world (Grace et al., 2012; Randolph et al., 2007). However, this dependence comes with a disproportionately high burden of zoonotic infections and leaves the health and livelihoods of many rural communities particularly vulnerable to incursions of animal diseases (WHO, Dfid, FAO, & OIE, 2006). Global urbanization is also causing increasingly frequent contact between wildlife, human and domestic animal populations, with resultant increases in zoonotic disease spillover from wildlife populations: 71.8% of recently identified emerging zoonotic disease events had a wildlife origin (e.g., severe acute respiratory syndrome, Ebola, Nipah), and such events have significantly increased in recent times (Jones et al., 2008). Animal rearing, slaughter, preparation and food consumption practices in many LMICs can further promote transmission of infectious animal and zoonotic diseases. Throughout Asia for example, live “wet” animal and seafood markets facilitate high‐density mixing of people and multiple domestic and wild animal host species from diverse geographical locations and can drive transmission, amplification and recombination of circulating viruses that may give rise to new strains with pandemic potential (Offeddu et al., 2016; Schar et al., 2019), including zoonotic swine‐ and avian‐origin influenzas and the more recent novel coronavirus SARS‐CoV‐2 (WHO, 2020b).

Accurate and rapid field diagnosis of infectious and zoonotic animal diseases in LMICs has many benefits. Early identification and management (e.g., treatment, isolation or culling) of infected animals can avert future medical costs associated with advanced disease in both animals and people, thereby benefiting health and livelihoods of subsistence farmers, communities and resource‐limited government services. From a global perspective, such early diagnosis and management decisions can prevent onward transmission of infectious pathogens, protecting the health and wellbeing of human and animal populations worldwide, and potentially containing epidemics that would otherwise have serious and far‐reaching consequences.

Few infectious diseases have pathognomonic clinical signs or syndromes, which makes presumptive diagnosis, treatment or management based on clinical appearance alone inadvisable. One study evaluated the diagnostic accuracy of Bangladeshi veterinarians identifying foot and mouth disease (FMD) and peste des petits ruminants (PPR) in cattle and goats based on clinical presentation and reported overall sensitivity and specificity of 54% and 81%, respectively, compared to polymerase chain reaction (PCR) testing (Haider et al., 2017). Unfortunately, improved diagnostic and surveillance capacity for animal diseases in many LMICs is severely hampered by a combination of factors, including under‐resourced field veterinary services, difficulties in sending samples to animal health laboratories (that may also be limited in the tests they can perform), and a general lack of investment by governments (WHO, Dfid, FAO, & OIE, 2006). Well‐meaning international donors donate specialized laboratory equipment and vehicles to government veterinary services that may not have sufficient budgets or resources to keep them adequately staffed, equipped or maintained (OIE, 2011). In many LMICs, long transport distances and unreliable sample referral logistics (e.g., poorly maintained roads and vehicles, fuel shortages, inadequate courier networks, seasonally inaccessible roads), difficulties maintaining cold chain, under‐equipped laboratories, shortages of trained personnel and prohibitively expensive operating costs contribute to long turnaround times between sampling, laboratory diagnosis and medical follow‐up. In Vietnam for example, suspected avian influenza samples take an average of 6–24 hr to reach a diagnostic laboratory, with total turnaround times from submission to result averaging 2.5 days (Inui et al., 2019). In Uganda, prior to the implementation of strengthening interventions, the median transport time of human diagnostic tuberculosis samples was 12 days (range 1 to 240 days), with only 9% of specimens reaching the central laboratory within the three‐day target transport time (Joloba et al., 2016). For highly infectious animal diseases of agricultural and zoonotic significance, such delays can have catastrophic outcomes. Rapid, on‐the‐spot diagnosis of infectious animal diseases in LMICs, therefore, could potentially avert or mitigate these constraints and consequences.

Point‐of‐care tests (POCTs) are defined as, “a fully or partially automated table‐top, portable or disposable device able to be operated in a non‐laboratory environment by non‐technical staff to deliver a same‐day, on‐site, clinically relevant, diagnostic test result” (Lehe et al., 2012). POCTs, also known as “rapid diagnostic tests”, “point of need tests” and “near patient tests”, come in a range of different formats, and are currently being used by human, animal and plant industries for a range of applications worldwide. They are designed to be portable, user‐friendly, simple to use, and usually have a turnaround time from sample to result in under an hour, allowing diagnosis and management decisions to be initiated within the same encounter. Many different POCT platforms and formats exist, ranging from paper‐based lateral flow assays (LFAs) and dipsticks like the globally recognized urine pregnancy test, to portable nucleic acid detection systems (e.g., loop‐mediated isothermal assays (LAMP), recombinase polymerase assays, portable and/or isothermal PCR devices), to handheld nanopore sequencing devices, wearable electronic sensors, “smart” textiles and more (Nayak et al., 2017; St John & Price, 2014; Vashist, 2017; Yager et al., 2008; Zarei, 2018). Some POCTs detect a single analyte or disease agent and others allow multiplexing to incorporate testing for two or more targets; some are disposable single‐use cartridges or cassettes, while others provide a portable platform on which a suite of different assays can be run. POCTs may be applicable to a range of different clinical purposes, including screening, diagnosis, monitoring, prognosis, surveillance or staging (WHO, 2019a). Many POCTs utilize smartphones, Wi‐Fi and/or Bluetooth connections to allow data transmission between remote field sites and central databases (Peeling & McNerney, 2014; St John & Price, 2014). POCTs typically require very small sample volumes, allowing minimally or non‐invasive sampling methods such as capillary blood sampling or swabbing techniques that may increase testing compliance in some cultural settings where invasive sampling methods such as venepuncture are poorly tolerated.

Recent years have seen POCT development and commercialization increase exponentially—a PubMed search for “point of care test” returned over 17,500 results at the time of writing—and the trend is predicted to continue as methodologies are refined and new technologies emerge. In the human medical space alone, the global market for diagnostic POCTs was valued at $24 billion USD in 2018 and is expected to more than double by 2026 (Reports & Data, 2019). While clinical chemistry POCTs dominate at present (Frost & Sullivan, 2016), infectious disease POCTs are predicted to grow at the fastest rate over the next 5–10 years, thanks in part to a number of high profile funding calls, mergers and collaborations in recent times (Reports & Data, 2019). The veterinary diagnostic POCT market is similarly predicted to increase, based largely on adoption of developments and technologies from the human POCT sector, coupled with increases in livestock ownership and per capita income, particularly in emerging Asian economies such as China and India (TechNavio Insights, 2014).

For infectious diseases, the biggest potential for POCTs is to significantly disrupt traditional laboratory‐based diagnostic pathways in LMICs, especially in decentralized rural settings where maintenance of cold chain and transport of field‐collected samples to the nearest capable laboratory is particularly challenging. When employed at critical disease transmission points, POCTs have the potential to prevent or curtail emerging or future epidemics, aid in contact tracing and facilitate mapping of the geographical distribution and evolution of infectious diseases in essentially real time. Studies demonstrating this potential in developing countries are increasing. Cepheid’s Ebola GeneXpert POCT, for example, which reduces the turnaround time for Ebola sample testing from days to hours, was credited by researchers and health officials as helping to contain the disease spread during a 2018 outbreak in the Democratic Republic of the Congo (Butler, 2018). Diagnostic POCTs could be used to great effect in live “wet” animal markets, allowing rapid identification of novel or emerging pathogens in domestic and wild animals as well as humans, enabling traceback of pathogens up the supply chain to determine the geographic location of disease emergence, and potentially providing essential early warning of disease spillover into susceptible human populations in high‐risk areas. POCTs could also be used effectively at border crossings, airports and other points of entry, where rapid identification of infected animals or animal products is crucial to prevent disease incursions beyond prescribed borders.

Despite the abundance of POCTs on the commercial market and clear role for POCTs in remote and rural settings, their true potential in LMICs is still far from being realized. To explore possible reasons for this gap, this literature review sought to investigate whether POCTs are currently being used in LMICs for diagnosis of infectious and zoonotic animal diseases, to determine characteristics of “ideal” POCTs that would facilitate their use and to identify any barriers to uptake in these settings.

## 2 MATERIALS AND METHODS

This narrative literature review used a hermeneutic approach that emphasized continuous engagement with and the gradual development of a large body of literature, to develop understanding and insights related to this broad and complex topic (Boell & Cecez‐Kecmanovic, 2014). The interpretation and critique that this narrative form of review would bring to this topic was preferred over a systematic approach more suited for addressing narrowly focussed research questions (Greenhalgh et al., 2018; Thorne, 2018). We used an iterative search strategy of electronic databases, including PubMed, Web of Science and Google Scholar, using different combinations of the following words and phrases to identify relevant publications: POC [point‐of‐care], “point of care tests”, POCT, “field test”, “rapid test”, “rapid diagnostic test”, zoonoses, infectious, veterinary, livestock, animal, “developing countries”, “low and middle income countries”, socioeconomic, impacts, acceptability, barriers and innovations. We also searched reference lists from key reviews and articles to identify additional publications of interest.

We did not attempt a formal, comprehensive systematic review of the literature due to the breadth and complexity of the topic, and the large variety in the type of reference materials examined. Nevertheless, we screened articles based on titles, abstracts and full texts, and purposively selected representative articles for inclusion in this review based on the following criteria:

- Inclusion criteria: Any publications relating to the testing, validation, review and commentary of diagnostic POCTs for infectious animal diseases (including zoonoses) in LMICs, published in English, in any year through and including January 2020. We selected studies that were relevant under the following categories: 1) usage, including reviews, trials and comparative studies of diagnostic infectious animal disease POCTs in LMICs; 2) considerations for aspects of the “ideal” POCT, with particular emphasis on applications in LMICs; 3) barriers to usage and uptake of infectious animal disease POCTs in LMICs.
- Exclusion criteria: Any publications involving non‐diagnostic POCTs, or POCTs for diagnosis of non‐infectious diseases in animals or of human‐only diseases; publications relating to non‐POCT animal diagnostic methods; any media in any languages other than English. Foreign language material was excluded because of the cost and time required for translation.

Where specific examples of publications regarding usage, implementation or impact of veterinary POCTs in LMICs were missing from the literature, we searched for relevant examples from the medical literature in order to provide a comparison for discussion. We also searched organizational websites including the World Organisation for Animal Health (OIE) and Food and Agriculture Organization of the United Nations (FAO) for information relating to POCTs for diagnosis of animal diseases. We also identified manufacturers of POCTs from key publications and documents and searched the internet for additional POCT manufacturers to identify those currently producing commercial diagnostic veterinary POCTs, and to obtain validation data and test kit inserts for infectious animal disease POCTs where available. The final bibliography included 567 documents (including journal articles, reports and guidelines) and 19 commercial POCT kit inserts from 11 manufacturers.

## 3 RESULTS

### 3.1 Are diagnostic POCTs currently being used for infectious animal diseases in LMICs? What are their impacts?

There have been increasing numbers of studies reviewing POCTs for diagnosis of infectious animal diseases published in recent years, and reports of their application in LMICs have also been on the rise. POCTs for rapid detection of infectious animal diseases with important zoonotic and/or economic impacts are the most commonly reported and often a range of different POCT formats have been developed, including for FMD (Abd El Wahed et al., 2013; Bath et al., 2020; Dukes et al., 2006; Madi et al., 2012; Reid et al., 2001; Yamazaki et al., 2013), highly pathogenic avian influenza (HPAI) (Boland et al., 2006; Imai et al., 2007; Postel et al., 2010; Slomka et al., 2012; Takekawa et al., 2010), canine rabies (Léchenne et al., 2016; Rupprecht et al., 2018; Tenzin et al., 2020) and ASF (Cappai et al., 2017; Sastre, Gallardo, et al., 2016; Sastre, Pérez, et al., 2016). Other target diseases for diagnostic veterinary POCTs include anthrax (Kurosaki et al., 2009; Muller et al., 2015; Pillai et al., 2019), PPR (Brüning‐Richardson et al., 2011; Rajko‐Nenow et al., 2019; Yang et al., 2017), bovine tuberculosis in cattle and in various wildlife species (Fresco‐Taboada et al., 2019; Lyashchenko et al., 2008; Tschopp et al., 2010), animal African trypanosomiasis (Boulangé et al., 2017) and a variety of parasites including *Anaplasma marginale* (Giglioti et al., 2019), *Trichenella* (Li et al., 2019) and *Haemonchus contortus* (Melville et al., 2014). Many of the published studies report the development and evaluation of these diagnostic POCTs, and although most remark on their potential to be used in remote and resource‐limited settings, few actually take the tests out of laboratory environment and into the field.

Some notable examples do exist in the literature. Field studies in eastern Africa used a commercially available, pan‐serotype‐specific PCR‐based assay for detection of FMD using lyophilized reagents and a portable, field‐ready thermocycler, and obtained diagnostic accuracy comparable to that of an OIE‐recommended laboratory‐based test (Howson et al., 2018). The POCT was further able to reliably detect different serotypes of FMD viral material in a variety of samples taken from pre‐clinical, clinical and clinically recovered cattle, with results available in under 90 min (Howson et al., 2018). A LAMP assay has also been developed and validated for rapid field detection of FMD, specifically designed to optimize the speed and operability of the test by non‐laboratory personnel on unextracted field samples (Bath et al., 2020). Loth et al. (2008) conducted field testing of two LFAs for detection of HPAI in oropharyngeal swabs taken from free‐ranging village chickens in Indonesia, and PCR testing on replicate swabs confirmed diagnostic sensitivities and specificities of the POCTs as 69%–71% and 98%, respectively. More recently, a Vietnamese pilot study reportedly took portable nucleic acid extraction and insulated isothermal PCR platforms into live bird markets to conduct rapid, on‐the‐spot testing of oropharyngeal swabs from poultry for detection of HPAI (Schar et al., 2019). A LFA for the detection of PPR virus in ocular and nasal swabs was trialled in field sites in Pakistan, Ethiopia, Ivory Coast and Uganda, and the test results obtained within 15–30 min reported diagnostic sensitivity and specificity of 84% and 95%, respectively, compared to PCR (Baron et al., 2014). The authors of this study also reported feedback from the field trials as being uniformly positive, with the portability of the tests and ease of use particularly emphasized.

Impacts of these POCTs on in‐country disease control or surveillance were not readily available from the literature.

### 3.2 What are the “ideal” characteristics for diagnostic infectious animal disease POCTs in LMICs?

#### 3.2.1 Fitness for purpose

While POCTs are designed to be simple and easy to use, their underlying biochemical processes are nevertheless highly sophisticated, and results need to be interpreted with due consideration of the tests’ fitness for purpose, including strengths, limitations and applications in various settings (Gardner et al., 2019). Estimates of diagnostic validity depend on several factors, including specimen type and quality, stage of infection (pre‐clinical versus clinical phase), strain of pathogen and pathogen load in the host, and host characteristics including age, sex, vaccination status, pregnancy and species (Gardner et al., 2019). POCTs that detect circulating host antibodies can only confirm an animal’s exposure to a pathogen rather than a current infection and may return false‐negative results if the samples were collected before a detectable immune response was developed. Pathogen‐derived nucleic acids may be detectable in animals that appear clinically well, allowing identification of early or late stages of infection when clinical symptoms are not yet present or have already disappeared. This is of particular relevance for chronic or sub‐clinical diseases. Sample contamination and non‐specific reactions to non‐target pathogens or matrix components may also lead to false‐positive results.

All these factors should be considered prior to the development of a new POCT. Like any diagnostic test, a POCT should, above all, be fit for its intended purpose; that is, it should meet clearly defined, pre‐decided criteria for its intended use (e.g., to confirm suspect or clinical cases of disease in an individual or defined population, to estimate prevalence of infection or exposure to facilitate risk analysis, to determine immune status of individuals or defined populations, to demonstrate freedom from infection in an individual or defined population, etc) and other desired attributes including characteristics of safety and efficacy (OIE, 2018a). An ASF POCT with low diagnostic sensitivity, for example, would be unsuitable as a standalone test for screening live pigs but may be sufficiently sensitive to be used as a herd test, or to confirm infection in individual pigs that died from ASF, in which large amounts of virus are present (Oura et al., 2013). Target product profiles (TPPs) outline the desired “profile” or performance characteristics of a new product (including diagnostics, drugs and vaccines) and are used by organizations such as the WHO, United States Food and Drug Administration (FDA), and Foundation for Innovative New Diagnostics (FIND) as planning tools to identify key test criteria, guide test development and set research and development (R&D) targets for funders and developers (FIND, 2019; WHO, 2019b). Ideally, TPPs for each novel POCT should be defined through several rounds of discussions with key stakeholders including disease experts, target users and manufacturers.

Once these characteristics have been established to ensure fitness for purpose, other considerations should also be addressed. The “ASSURED” criteria set out by the World Health Organisation (WHO) state the ideal characteristics for a field‐ready diagnostic test as Affordable, Sensitive (few false negatives), Specific (few false positives), User‐friendly (able to be performed in a few steps with minimal training), Robust (no cold storage needed) and rapid (results available in under 30 min), Equipment‐free and Deliverable to those who need it (Kosack et al., 2017). Social studies have indicated that shorter diagnostic turnaround times (up to 20 min), high diagnostic accuracy (sensitivity and specificity above 90%), low cost and ease of use are particularly important factors for some public health workers (Asiimwe et al., 2012; Hsieh et al., 2011). It should be emphasized that there can be no “one size fits all” description of the ideal diagnostic POCT, as fitness for purpose will be determined by the needs, intentions and resources of the veterinary services in each distinct geopolitical setting.

#### 3.2.2 Additional considerations for LMICs

To be truly deliverable to remote locations, POCTs should be portable, self‐contained and either equipment‐free or battery‐operated, with thermostable, lyophilized reagents that do not require cold chain or reconstitution with high‐quality solutions. As target users of POCTs are expected to have minimal laboratory training, POCT protocols should require minimal preparatory or extraction steps prior to sample testing (Crowther et al., 2006; Pai et al., 2012). POCT devices would ideally include internal quality controls, with out‐of‐range results clearly identifiable to POCT operators.

POCTs that have multiplexing ability, enabling samples to be tested against several diseases in parallel, would be expected to significantly improve acceptance by farmers and livestock owners. Notable examples of multiplexed veterinary diagnostic POCTs include LAMP assays that detect all seven distinct FMD serotypes (Yamazaki et al., 2013), and an in‐development LFA for the simultaneous testing of ASF and classical swine fever viruses in pig blood (Sastre, Pérez et al., 2016). POCTs able to differentiate infected from vaccinated animals, for example non‐structural protein FMD LFAs (Bionote, 2020; King et al., 2015) would also be instrumental for monitoring the progress of disease elimination or eradication campaigns, such as the OIE’s mission to eradicate PPR by 2030 (OIE, 2019).

Cost and cost‐effectiveness are important factors to consider when evaluating POCTs for potential introduction into LMICs. While costs of individual tests can be higher for POCTs compared to high‐throughput laboratories, POCTs are likely to appeal to government veterinary services in LMICs due to their lower initial purchase price, as well as decreased ongoing costs for operation, maintenance and personnel training. Modifying testing strategies where appropriate can provide additional savings, such as pooling of samples to reduce numbers of test runs; this was successfully implemented in Vietnamese live poultry markets, in which oropharyngeal swabs from five birds were pooled for testing with HPAI POCTs, at a cost of $10 USD per test run (Schar et al., 2019). Other considerations include costs of manpower, equipment and reagent storage, and transport for veterinary staff implementing POCTs in the field. Indirect costs include effects of POCTs on policy, trade and the economy, as well as on public health, welfare and trust (Greiner & Gardner, 2000).

POCTs can also provide interim diagnostic solutions while longer‐term laboratory capacity is being developed. Portable, field‐ready LAMP and PCR platforms, for example, are currently being used for animal disease testing in Timor‐Leste while the country’s PCR laboratory capability is progressing [personal observation, JA]. However, it should be stated that POCTs are rarely intended to replace traditional laboratory testing altogether, as representative samples will typically still require laboratory testing to confirm and genetically characterize the disease agent, particularly for outbreaks of notifiable or reportable diseases. Effective sample transport networks and suitably equipped and staffed laboratories will therefore still be needed in LMICs for the foreseeable future (Dowdy, 2016; Fonjungo et al., 2017). In developed countries such as Australia, POCTs are being used as frontline surveillance tools for preliminary diagnosis of important livestock diseases including FMD and ASF, with the condition that all samples, including those that test negative by POCTs, will continue to be tested in accredited state laboratories using OIE‐approved “reference standard” methods (Bath et al., 2020).

### 3.3 What are the barriers for usage and uptake of infectious animal disease Pocts in LMICs?

#### 3.3.1 Lack of validated and affordable diagnostic POCTs for infectious animal diseases

While some commercially available POCTs for diagnosis of infectious animal diseases have been described above, there are still many pathogens for which no rapid, field‐ready tests are available. The R&D process for a new diagnostic (human) test costs $2–10 million USD and takes 5–10 years (Peeling & Mabey, 2010). Products are market driven, thus more POCTs are available for those diseases that are commonly seen in developed nations, where affordability is not an issue. Neglected zoonotic and agricultural diseases affect mainly impoverished, marginalized communities that have little purchasing potential for novel diagnostic tests (WHO, Dfid, FAO, & OIE, 2006), making them unattractive product targets for commercial investors. New test development is largely supported by a handful of public and philanthropic organizations, but investment is often insufficient and unevenly distributed: of the $3.5 billion USD invested in R&D for neglected diseases in 2017, 70% was allocated to HIV/AIDS, tuberculosis and malaria (Chapman et al., 2019; Moran, 2011), and as of July 2019 less than 0.5% of global health‐related products in the development pipeline were targeting neglected tropical diseases (WHO, 2019b).

Test developers in LMICs may be more motivated to invest in POCT R&D for animal diseases that are prevalent in their regions, however may be hampered by a lack of funding, personnel and resources, or global recognition of POCTs if developed.

#### 3.3.2 Lack of mandated central register of approved POCTs for detection of infectious animal diseases

The WHO publishes R&D Blueprints for priority human diseases in which ideal product specifications for POCTs are described (WHO, 2020a). They also provide a service for the evaluation of novel POCTs for a range of human diseases, with favourably evaluated assays included in their widely used “model list of essential in vitro diagnostics” (WHO, 2018, 2019a). The OIE offers a formal validation and certification process for diagnostic animal disease tests, including (but not specifically) POCTs, and also maintains a register of diagnostic veterinary kits that have been certified as fit for purpose (https://www.oie.int/scientific‐expertise/registration‐of‐diagnostic‐kits/the‐register‐of‐diagnostic‐kits/). The assessment process takes an estimated 135 days and costs 4,500 Euros. While generally accepted as “best practice” for veterinary diagnostics, as of August 2020 only 14 kits are registered for a total of 11 pathogens, and only two are POCTs. Because this process is not mandatory, and because the register is not widely used, manufacturers—particularly smaller, underfunded POCT manufacturers in LMICs—may see little value in registering. This lack of a comprehensive, widely used reference point for approved diagnostic animal disease POCTs makes it difficult for LMICs and other users to differentiate reliable POCTs from cheaper, unvalidated tests that are entering the market at present. Without reliable evidence of a test’s diagnostic accuracy or fitness for purpose, many LMICs may simply be selecting POCTs based on purchase price alone.

#### 3.3.3 Current limitations to POCT validation and regulatory processes

##### Lack of consistency and transparency of POCT validation data

The OIE Terrestrial Manual has a section outlining the recommended validation pathway for novel diagnostic tests for infectious animal diseases (OIE, 2018c), including assessment of analytical and diagnostic characteristics, repeatability and reproducibility, for independent evaluation of test performance under varying environmental and operator conditions. There are separate chapters specifically addressing the development and optimization of antibody, antigen and nucleic acid detection assays; while generally comprehensive documents, none of these extend to or include requirements for those assays to be used in field settings (i.e., POCTs). Several other checklists and guidelines have been created for validation of diagnostic assays and to facilitate transparency of reporting diagnostic accuracy studies (Cohen et al., 2016; Huddy et al., 2015; Shabir, 2004; Whiting et al., 2011). In some countries, such as Germany (FLI, 2019), POCTs for the detection of notifiable and reportable animal diseases must be evaluated and approved by the national licensing authority. For most countries however, adherence with “best practice” guidelines for manufacturers of animal disease POCTs is neither mandatory nor regulated.

Without regulations enforcing test validation, animal disease diagnostics—including POCTs—of varying quality and effectiveness can be sold and used, often in the developing world, with limited, misleading or incorrect data about their diagnostic validity in the target population (Peeling & Mabey, 2010). Indeed, some disturbing trends are observable from available literature. Studies reporting diagnostic test accuracy in general often fail to transparently and completely describe essential information about core elements including study design (e.g., differences between the study and target population, and patient selection), and tend to be overly generous and optimistic about tests’ value (Cohen et al., 2016). POCT kit inserts generally include some validation data obtained from small, carefully regulated laboratory studies, which are unlikely to represent real‐world conditions, and while diagnostic sensitivity and specificity values are often stated, they rarely include confidence intervals which would provide end users with data about sample sizes or test power. Positive test controls are often negative samples “spiked” with pathogenic material at concentrations higher than in clinical samples and may not include typical sample matrices or common contaminants (e.g., blood, mucus, soil), thereby artificially inflating test performance (Crowther et al., 2006). Host‐pathogen interactions may be different in experimental animal species and breeds (e.g., Landrace pigs, commercial layer chickens) than in native animal populations. Experience from the field showed that levels of Newcastle disease virus shed in native Bhutanese chickens are detectable by POCTs in oropharyngeal secretions but not in faeces, yet commercial POCT kit inserts recommend testing on both sample types [personal observation, RBG]. Testing panels of positive and negative samples typically represent disease prevalence far above or below what would be expected in natural populations, which can also artificially inflate indicators of test accuracy (Banoo et al., 2006; Dowdy et al., 2011). Manufacturer quoted predictive values are likely to be inflated by artificially high prevalence in validation test populations, meaning the tests will produce more false positives in real‐world settings where disease prevalence is usually lower (Crowther et al., 2006). Many POCT users with limited diagnostic and epidemiological experience will not have the knowledge or understanding to correctly interpret and apply these data to their own environments; consequently, regulations should be developed for manufacturers to standardize the validation data published in test kit literature.

##### Lack and limitations of field validation studies for infectious animal disease POCTs

Typically, POCTs are utilized in the field under varying environmental conditions, on a range of sample types collected in non‐sterile settings by operators with a diverse range of experience, training and proficiency. Storage conditions of POCT devices and reagents may be subject to a higher variability than in an accredited laboratory. Consequently, in addition to general validation requirements for diagnostic assays, POCT‐specific parameters such as robustness and ruggedness need to be defined and addressed during the validation process.

Intra‐ and inter‐operator variation need to be assessed in POCT validation studies under a range of realistic field conditions that are representative for each intended target location. For example, if a test has been validated for use with oropharyngeal swabs but field operators are only able to take cloacal swabs, the results obtained for these specimens may not be reliable. Experience and competence of operators, as well as variations in sample types and quality, and environmental factors including exposure to direct sunlight, humidity, temperature, dust, soiling and other physical impacts are all key factors that will impact on a POCT’s fitness for purpose. Results will help to detect robustness against internal variation (repeatability), and ruggedness against external conditions such as climatic conditions and levels of proficiency (reproducibility). To assess and monitor reliability, it is important to include internal quality controls to confirm the basic functioning of the device; for example a weak positive control (to confirm sensitivity of device and avoid false‐negative results); a negative control (to confirm that test reagents are not contaminated producing false‐positive results); and for nucleic acid detection assays, an internal control to identify the presence of matrix inhibitors.

With some notable exceptions, including some described earlier (Bath et al., 2020; Certoma et al., 2018; Howson et al., 2018), many validation studies fail to conduct actual field testing of novel POCTs. Outside the laboratory environment, suboptimal testing conditions including variations in temperature, humidity, operator ability, water and reagent quality, inadequate cold chain, and poor or non‐existent quality assurance systems can all contribute to lower test accuracies than those reported by POCT manufacturers. Diagnostic sensitivities of six commercially available LFAs for detection of rabies virus in brain tissue, for example, reportedly ranged from 0% to 100% for field samples, with a maximum sensitivity of 32% for samples taken from experimentally infected animals (Eggerbauer et al., 2016). The authors of that comparative study also noted that POCT kit instructions failed to specify the requisite volume and collection locations of brain tissue, which could substantially affect test results (Eggerbauer et al., 2016). One of the rabies POCTs was subsequently improved (diagnostic sensitivity and specificity approaching 100%) by omitting the first dilution step recommended by the manufacturer (Léchenne et al., 2016), suggesting the assay’s development and validation processes may not have been suitably rigorous.

Any application of a POCT outside the strict workflow in which it was validated (including sample types or non‐target host species) can cause incorrect and unspecific reactions that could affect treatment and management decisions and may undermine user confidence in POCTs. Several LFAs for detecting rabies in brain material showed better diagnostic performance on samples from South Africa than from Eurasia and northern America (Eggerbauer et al., 2016), which could reflect differences in viral strains, sample preparation methods or a range of other factors. Some HPAI POCTs demonstrated higher diagnostic sensitivities with samples from chickens (65%–85%) than from ducks (33%–53%) (Slomka et al., 2012). Accuracy of test results may also depend on strict sample collection, extraction and/or storage requirements, including maximum storage times. Rapid FMD PCR assays trialled in eastern Africa, for example, showed reduced diagnostic accuracy in field samples taken from older (four days and older) than from fresh (one‐ to three‐day‐old) lesions (Howson et al., 2018). One rabies LFA requires brain samples to be tested immediately after collection (Léchenne et al., 2016), while another ASF LFA allows blood, collected into any anticoagulant, to be refrigerated for up to four days prior to testing (Ingenasa, 2019). Despite the importance of these factors to end users, data about POCTs’ validation pathways, applicability to other species and sample types, and general fitness for purpose in field settings is largely lacking.

##### Difficulties conducting full validation for some infectious animal disease POCTs

Some situations exist in which full independent validation of novel diagnostic POCTs is not feasible. During the early stages of the 2013–2016 west African Ebola epidemic, for example, there were no validated devices available for rapid disease diagnosis. However, emergency use authorizations issued by the US FDA enabled several POCTs that had undergone initial validation to be used in the field, thus helping to contain the spread of Ebola during subsequent outbreaks (Butler, 2018; Dhillon et al., 2018). Similarly, several countries’ authorities, including Australia, have granted emergency approval for use of POCTs detecting SARS‐CoV‐2 during the current COVID‐19 pandemic, including Cepheid’s GeneXpert nucleic acid test and a number of LFAs (TGA, 2020).

Disease investigation in wildlife presents unique challenges, due to often limited data on wildlife physiology and pathogen behaviour, as well as logistic and regulatory considerations around collection, use and international transport of wildlife and their tissues (OIE, 2018b). A recent review from Jia et al. (2020) about validation of laboratory tests for infectious diseases in wild mammals revealed incomplete or absent information about sampled animals and/or species, case definition criteria and source and target populations that would impact test validity and inform applicability of test results, including their status as naturally infected captive, free‐ranging or experimentally challenged animals. Sampling is often opportunistic, and individual capture and follow‐up frequently unachievable. Given these limitations, the OIE provides guidelines for provisional validation of diagnostic wildlife disease tests, which can provide confidence in results (OIE, 2018b). An extension of this was the development of a network approach for provisional validation of Hendra virus laboratory diagnostics due to limited numbers of positive samples (Colling et al., 2018); this may also be successful for validation of wildlife diagnostic POCTs, and/or for POCT developers in disease‐free regions where import restrictions obstruct access to viable samples for validation studies. Australian biosecurity laws, for example, prohibiting the importation of live FMD virus prompted Bath et al (Bath et al., 2020) to conduct preliminary validation of a novel FMD LAMP assay on inactivated FMD samples in Australia, followed by field testing of live samples in Bhutan and Thailand where the disease is endemic.

### 3.4 Socioeconomic considerations for animal disease Pocts at the community level

Early identification and management (treatment, isolation or culling, for example) of infected animals can avert future medical costs associated with advanced disease in both animals and people, thereby protecting the livelihoods and wellbeing of subsistence farmers and their communities and reducing burdens on resource‐limited medical and veterinary services. Increased empowerment of farmers with regard to animal treatment and management decisions may particularly benefit women and children, who are the primary carers of pigs, poultry and small ruminants in many developing countries (Donadeu et al., 2019), provided they are also empowered to spend money on their animals. For test results to translate into tangible benefits for communities, robust reporting and capable veterinary follow‐up must support the outcomes of POCTs, otherwise positive test results will be of little benefit to livestock owners, who may prefer to spend their money on antibiotics or other potentially curative treatments. The capability of veterinary services to conduct and follow‐up from POCTs in field settings will be determined by a range of factors, including workloads, training and the availability and sustainability of supplies (Osorio et al., 2018), and a breakdown in any of these areas may undermine community perception of the value of POCTs. In situations where test‐positive animals will lose market value (such as pigs who test positive for porcine cysticercosis (Hobbs et al., 2018)), require confirmatory testing and/or culling, owners must be suitably compensated. Failure to adequately compensate animal owners is likely to decrease compliance with testing or reporting of unwell livestock, and may lead to the unauthorized movement, hiding or salvage selling of these animals, potentially exacerbating disease transmission and undermining disease control efforts (Fèvre et al., 2006). Compensation for testing, and devalued or culled animals, however, is highly variable and entirely dependent on animal health policies of each country.

Target users of POCT in LMICs are typically not laboratory personnel and are likely to have limited capacity building opportunities necessary for using, interpreting and applying results of diagnostic tests (Nichols, 2007). Furthermore, POCTs are likely to be used in remote field sites, where experienced personnel for supervision or training of POCT users may be in short supply. Responsibility for interpreting POCT results and instigating treatment or management decisions may vary, based on the available staff resources in each area, and on the nature of the disease or pathogen being tested. Testing for notifiable diseases or pathogens of pandemic potential, for example, may increase the level of oversight or confirmatory testing needed. Technology could be leveraged to assist in remote sites, or in areas where technical personnel are lacking; some POCTs have inbuilt connectivity such as Wi‐Fi and Bluetooth, and smartphones can be used to photograph test results, allowing information to be transmitted and received between remote field sites and central veterinary offices or laboratories.

Other social considerations include community perception and acceptability of POCTs, which can influence POCT uptake in different settings. Communities may have traditional or cultural beliefs that make them averse to particular sampling methods, such as needle use in pigs (Hobbs et al., 2020). A lack of trust in a test’s accuracy, or misperceptions about the extent of a test’s scope can undermine success of POCT implementation, as demonstrated in social studies evaluating the use of malaria POCTs in human health settings: *malaria treatment was reportedly often prescribed despite a negative result* (Johansson et al., 2016) and *…respondents also believed the tests could identify any cause of illness, beyond malaria* (Ansah et al., 2013). Without community support, even fully validated and effective diagnostic POCTs are unlikely to be successfully implemented in animal disease control or surveillance programs in LMICs.

## 4 DISCUSSION

Economic impacts of infectious animal and zoonotic diseases can be severe and far‐reaching, as has become all too apparent during the global COVID‐19 pandemic—a zoonotic virus that is widely accepted to have a wildlife origin (Zhou et al., 2020). The current Asian ASF epidemic is causing further heavy losses to agricultural and trade sectors, and impacting the daily lives of millions of consumers, livestock and crop producers and policymakers worldwide (Hamaide, 2019; ProMED‐mail, 2019). Social impacts can also be substantial, although complex and difficult to quantify. Even in large‐scale outbreaks of non‐zoonotic animal diseases such as FMD and ASF, psychological distress can be experienced on individual, household and community levels due to uncertainty about income and livelihoods, witnessing or involvement in mass livestock culling, social isolation due to movement restrictions, loss of trust in authority, altered relationship dynamics and a loss of social cohesion (Buetre et al., 2013; Mort et al., 2005). The 2001 FMD outbreak in the United Kingdom has been described as *a traumatic and devastating experience for all those who were affected by it…a national crisis…probably one of the greatest social upheavals since the war* (Mort et al., 2005). Misdiagnosis of zoonotic diseases can further impact individuals, families and communities, potentially leading to fear, stigma and a loss of rights, such as for patients incorrectly diagnosed with Ebola who are unable to be cared for by family members, and who in the event of their deaths are unable to be buried according to traditional cultural practices (Pellecchia et al., 2015).

POCTs have the potential to significantly disrupt traditional laboratory‐based diagnostic pathways, especially in remote decentralized settings where sample referral networks and adequately equipped laboratories are particularly lacking. Diagnostic medical and veterinary POCTs are being used in LMICs and show clear benefits for disease diagnosis and surveillance, particularly when supported by policymakers (Mabey et al., 2012). However, despite the abundance of POCTs on the commercial market and their clear benefits in remote and rural settings, the potential benefits of these tests in LMICs remain largely unrealized.

Inadequate regulatory guidance and poor industry oversight has led to a proliferation of POCTs of varying quality and fitness for purpose released onto the market, presenting challenges to potential end users who are, by design, expected to have limited diagnostic experience. Accurate, independent test validation data for commercial POCTs are often incomplete or absent. Even after robust initial validation testing, POCTs that demonstrate excellent diagnostic performance in the laboratory can show markedly lower accuracy under field conditions, for a variety of reasons. Similarly, incorporation of POCTs in diagnostic pathways and disease testing algorithms that are successful in one setting may not have adequate uptake in others due to the varying and complex interplays between political, sociocultural and geographic factors, among others. Government veterinary services in LMICs should be aware of the costs, impacts, cost‐effectiveness and operational feasibility of incorporating POCTs into diagnostic workflows in their specific country context, and invest their resources accordingly. However, the lack of clear POCT‐specific validation guidelines promoted by an independent organization such as the OIE, the absence of a mandated or widely used centralized register of approved POCTs, and the dearth of independently validated field studies in the literature at present means that many LMICs may simply be selecting POCTs based on purchase price alone—a practice which may be creating more problems than solutions.

Urgent action is needed. Public and philanthropic funding agencies need encouragement to engage with stakeholders for development of TPPs, and to invest in the subsequent development and independent validation of targeted animal disease POCTs for use in field settings, especially for diseases that are only present in developing countries and consequently for which commercial manufacturing interest is low. The international community should be urged to adopt regulatory frameworks for the manufacture and commercialization of diagnostic POCTs, and to mandate transparent publication of comprehensive, independent POCT validation data about test accuracy and fitness for purpose. Standardized technical guidance and training should be made available for POCT users and policymakers in the developing world, with advice for evaluating POCT characteristics based on manufacturer information and available literature, conducting in‐house field validation and verification of diagnostic POCTS, and correctly interpreting and applying POCT results according to the relevant clinical setting.

Without government and community support, even the most accurate and cost‐effective diagnostics will fail to have any substantial or sustained impact in LMICs. Sociological research and user needs analyses are required to understand the drivers of animal disease transmission in LMICs, to investigate local knowledge, attitudes and practices relating to animal disease diagnosis and surveillance, to identify barriers and facilitators to POCTs use in regional contexts and to develop strategies for implementing POCTs in the most impactful way to benefit animals, people and communities. Even with reliable access to properly validated, fit for purpose POCTs, for diseases of high importance there will still be a need for confirmatory laboratory diagnosis and pathogen typing in LMICs for the foreseeable future. Therefore, support should also be given to initiatives that strengthen national veterinary and laboratory capacity, such as the Performance of Veterinary Services and laboratory twinning programs supported by the OIE and other international partners. Advances in and increasing accessibility to technology should also be utilized, for example using drones to assist with sample transport in remote locations (Mitchell, 2014; MSF, 2014) and dried tube specimens for proficiency testing in resource‐constrained settings (Parekh et al., 2010).

Infectious animal and zoonotic diseases have been increasing in incidence in recent times, particularly in LMICs with high human and animal density (Bordier & Roger, 2013), and are increasingly posing global threats. Rapid, on‐the‐spot diagnosis of infectious pathogens using POCTs can play an important role in containing these outbreaks, however concerted action must be taken by industry, governments, regulators and key stakeholders to overcome the challenges identified in this review in order to realize their potential benefits to animal and human populations worldwide.

## CONFLICT OF INTEREST

The authors have no conflicts of interests to declare.

## ETHICAL APPROVAL

The authors confirm that the ethical policies of the journal, as noted on the journal’s author guidelines page, have been adhered to. No ethical approval was required as this is a review article with no original research data.

## Data Availability

Data sharing is not applicable to this article as no new data were created or analysed in this study.
